# Supplementary material for: Synthesis of lipid-linked precursors of the bacterial cell wall is governed by a feedback control mechanism in Pseudomonas aeruginosa
Source: Nat Microbiol. 2024 Feb 9;9(3):763–75. doi: 10.1038/s41564-024-01603-2 (PMC10914600; doi:10.1038/s41564-024-01603-2)
Supplement: Supplementary file 1 — Supplementary Figs. 1–3, and Tables 1, 2 and 4. [file 41564_2024_1603_MOESM1_ESM.pdf]

nature microbiology

Article https://doi.org/10.1038/s41564-024-01603-2

Synthesis of lipid-linked precursors of the bacterial cell wall is governed by a feedback control mechanism in *Pseudomonas aeruginosa*

In the format provided by the authors and unedited

SI Table 1. List of suppressing MraY variants

Residue	Changed to
K14	E
Y21	H
L22	P
T23	A, P
L31	P
L160	R
M164	R
K166	E
Y217	H
G220	S
F224	S
A225	V
Y227	C
L228	P
G248	C
Q286	R
V289	M
I339	V
V347	M
L354	P
K358	M

SI Table 2: Cryo-EM data collection, refinement, and validation statistics

	***Ec*MraY** (EMDB- EMD-41373) (PDB 8TLU)
**Data collection and processing**	
Microscope	FEI Titan Krios
Magnification	105,000
Voltage (kV)	300
Electron exposure (e–/Å^2)	60
Defocus range (μm)	-1 to -2.5
Pixel size (Å)	0.832
Symmetry imposed	C1
Initial particle images (no.)	3,885,223
Final particle images (no.)	287,765
Map resolution (Å) FSC threshold: 0.143	3.8
**Refinement**	
Software	PHENIX 1.19.2
Initial model used (PDB code)	8G01
Resolution of unmasked reconstructions (Å, FSC=0.5)	4.1
Resolution of masked reconstructions (Å, FSC=0.5)	3.8
Correlation coefficient (CC_{mask})	0.76
Model composition	
Atoms (Hydrogens)	11180 (5703)
Protein residues	698
Ligands	0
B factors (Å^2) (min/max/mean)	
Protein	20.34/79.77/41.69
Ligand	-
R.m.s. deviations	
Bond lengths (Å)	0.003 (0)
Bond angles (°)	0.536 (0)
**Validation**	
MolProbity score	1.43
Clashscore	7.87
Poor rotamers (%)	0
Ramachandran plot	
Favored (%)	98.40
Allowed (%)	1.60
Disallowed (%)	0

SI Table 4. Bacterial strains used in this study

Strain	Description[*]	Reference
E. coli		
DH5α	Host strain for plasmid cloning	Invitrogen
BL21(DE3)	Expression strain for protein production	[1]
CAM333	C43(DE3) Δ*ponB*, Δ*pbpC*, Δ*mtgA*	[2]
LSM9	C43(DE3) Δ*pbpC*, Δ*mtgA fhuA*-	This study
Δ*slyD* BL21(DE3)		[3]
MG1655	Wild-type	[4]
MM119	Δ*ponA ponB(*E313D) Δ*lpoB*	[5]
P. aeruginosa		
PAO1	Wild-type	S. Lory
PA686	Δ*ponB* Δ*lpoA*	[6]
PA760	Δ*ponB* Δ*lpoA mraY*(T23P)	This study
PA662	Δ*PA0615–0628*; R2 pyocin deletion	S. Lory

References

1. Studier, F. W. & Moffatt, B. A. Use of bacteriophage T7 RNA polymerase to direct selective high-level expression of cloned genes. *J Mol Biol* **189**, 113–130 (1986).

2. Meeske, A. J. *et al.* SEDS proteins are a widespread family of bacterial cell wall polymerases. *Nature* **537**, 634–638 (2016).

3. Orta, A. K. *et al.* The mechanism of the phage-encoded protein antibiotic from ΦX174. *Science* **381**, (2023).

4. Guyer, M. S., Reed, R. R., Steitz, J. A. & Low, K. B. Identification of a Sex-factor-affinity Site in E. coli as gamma delta. *Cold Spring Harb Symp Quant Biol* **45**, Pt 1:135-140 (1981).

5. Markovski, M. *et al.* Cofactor bypass variants reveal a conformational control mechanism governing cell wall polymerase activity. *Proc National Acad Sci* **113**, 4788–4793 (2016).

6. Greene, N. G., Fumeaux, C. & Bernhardt, T. G. Conserved mechanism of cell-wall synthase regulation revealed by the identification of a new PBP activator in Pseudomonas aeruginosa. *Proc National Acad Sci* **115**, 3150–3155 (2018).

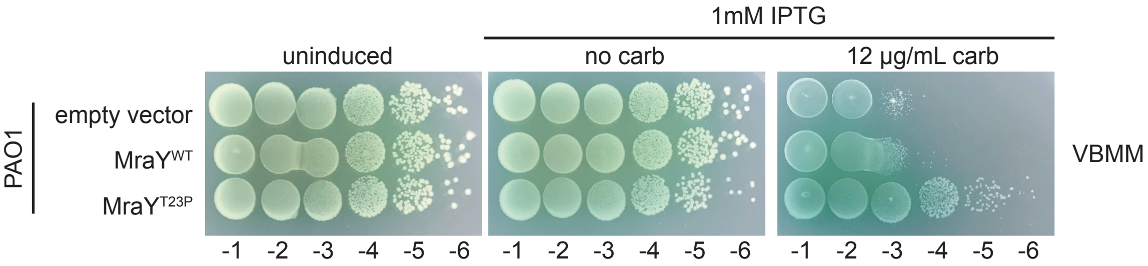

Supplementary Figure 1: Expression of MraY(T23P) promotes growth of *P. aeruginosa* on carbenicillin. Ten-fold serial dilutions of *P. aeruginosa* cells harboring expression plasmids producing the indicated MraY protein. Dilutions were plated on VBMM with or without IPTG to induce the MraY protein as indicated, containing carbenicillin at the concentration indicated. Abbreviations: WT, wild-type; VBMM, Vogel-Bonner minimal medium; IPTG, isopropyl-B-D-1-thiogalactopyranoside.

Supplementary Figure 2

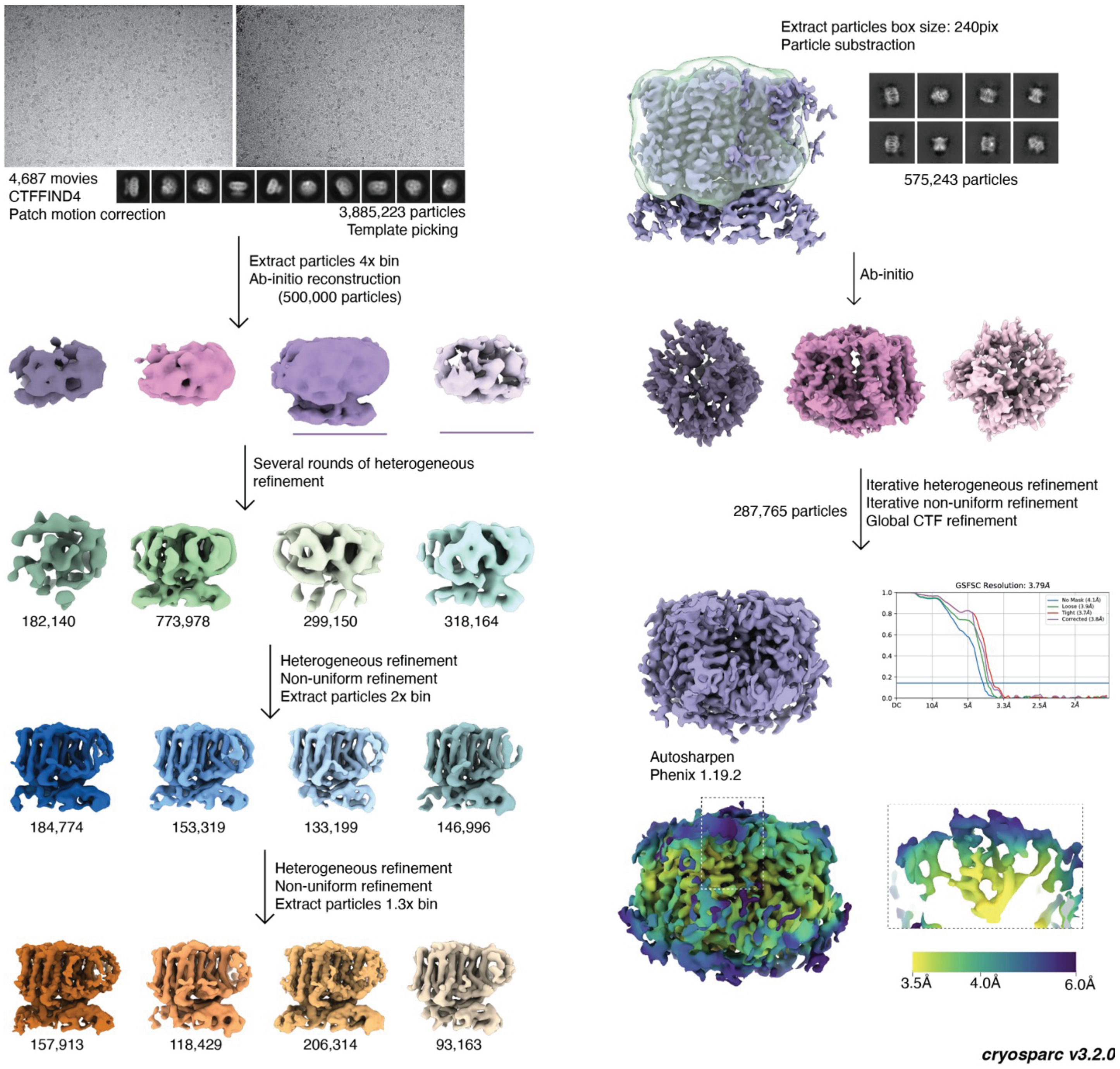

Supplementary Figure 2. Cryo-EM structure of ^{Ec}MraY(T23P) in the YES complex. Data processing was performed using cryosparc (v3.2.0). Representative movies are shown (top left) with corresponding 2D classes observed in the dataset. Arrows denote the methodology order, following several rounds of heterogeneous refinement. The number of particles sorted is shown below the densities. The masked volume of MraY (green, top right) used for particle subtraction is shown overlayed with the density (purple) of the entire YES complex. The final model is colored by resolution using the viridis color scheme. The unmodeled density at the dimer-interface is isolated for clarity and shown in a dotted box.

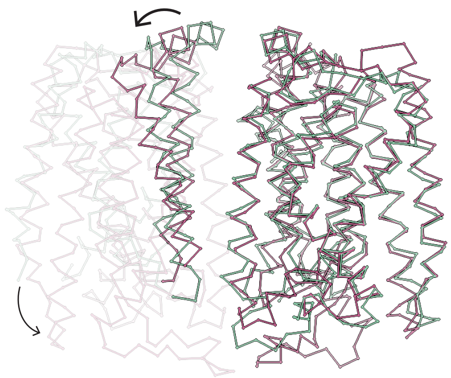

Supplementary Figure 3. Altered conformation of MraY dimers in the YES complex versus ^{Aa}MraY. View from the plane of the membrane. Stick representation of the α-carbon chain of ^{Ec}MraY(T23P) (pink) structurally aligned to ^{Aa}MraY (PDBID:4J72)(green). Molecules are aligned to the right chains in the figure. Arrows highlight the differences in ^{Aa}MraY compared to ^{Ec}MraY(T23P).
