# Supplementary material for: Synthesis of lipid-linked precursors of the bacterial cell wall is governed by a feedback control mechanism in Pseudomonas aeruginosa
Source: Nat Microbiol. 2024 Feb 9;9(3):763–75. doi: 10.1038/s41564-024-01603-2 (PMC10914600; doi:10.1038/s41564-024-01603-2)
Supplement: Supplementary file 2 — Reporting Summary [file 41564_2024_1603_MOESM2_ESM.pdf]

Corresponding author(s): Thomas G. Bernhardt
Last updated by author(s): 12/23/2023

Reporting Summary

Nature Portfolio wishes to improve the reproducibility of the work that we publish. This form provides structure for consistency and transparency in reporting. For further information on Nature Portfolio policies, see our Editorial Policies and the Editorial Policy Checklist.

Statistics

For all statistical analyses, confirm that the following items are present in the figure legend, table legend, main text, or Methods section.

n/a	Confirmed	
☐	☒	The exact sample size (*n*) for each experimental group/condition, given as a discrete number and unit of measurement
☐	☒	A statement on whether measurements were taken from distinct samples or whether the same sample was measured repeatedly
☐	☒	The statistical test(s) used AND whether they are one- or two-sided<br>*Only common tests should be described solely by name; describe more complex techniques in the Methods section.*
☒	☐	A description of all covariates tested
☒	☐	A description of any assumptions or corrections, such as tests of normality and adjustment for multiple comparisons
☐	☒	A full description of the statistical parameters including central tendency (e.g. means) or other basic estimates (e.g. regression coefficient) AND variation (e.g. standard deviation) or associated estimates of uncertainty (e.g. confidence intervals)
☐	☒	For null hypothesis testing, the test statistic (e.g. *F*, *t*, *r*) with confidence intervals, effect sizes, degrees of freedom and *P* value noted<br>*Give P values as exact values whenever suitable.*
☒	☐	For Bayesian analysis, information on the choice of priors and Markov chain Monte Carlo settings
☒	☐	For hierarchical and complex designs, identification of the appropriate level for tests and full reporting of outcomes
☒	☐	Estimates of effect sizes (e.g. Cohen's *d*, Pearson's *r*), indicating how they were calculated

Our web collection on statistics for biologists contains articles on many of the points above.

Software and code

Policy information about availability of computer code

Data collection	SerialEM Version 4.1 (CryoEM)
Data analysis	Statistical analyses were performed by Prism 9 (GraphPad Software, LLC.). MD simulations were run in Gromacs 2021.3. Images were made in VMD. Kinetic analysis of protein-lipid interactons and binding site identification were performed using PyLipID. Density and contact analyses of atomistic MD simulations were performed using MDAnalysis. LC/MS data were analyzed using Agilent MassHunter Workstation Qualitative Analysis software. Structural data were visualized using ChimeraX 1.6.1. The final map was generated using PHENIX-1.19.2. Data processing was performed using cryosparc V3.3.2. The contrast transfer function (CTF) was estimated using CTFFIND4 4.1.10 .

For manuscripts utilizing custom algorithms or software that are central to the research but not yet described in published literature, software must be made available to editors and reviewers. We strongly encourage code deposition in a community repository (e.g. GitHub). See the Nature Portfolio guidelines for submitting code & software for further information.

Data

Policy information about availability of data

All manuscripts must include a data availability statement. This statement should provide the following information, where applicable:

- Accession codes, unique identifiers, or web links for publicly available datasets
- A description of any restrictions on data availability
- For clinical datasets or third party data, please ensure that the statement adheres to our policy

All bacterial strains and plasmids developed in this study are available upon request. The atomic coordinates presented in this study have been deposited in the RSCB Protein Data Bank (PDB) under the accession number PDB: 8TLU. Previously published structures referenced in this manuscript can be accessed in the PDB using the accession numbers: 8G01 (E. coli MraY), 4J72 (A. aeolicus MraY), 5JNQ (E. boltae MraY).

Research involving human participants, their data, or biological material

Policy information about studies with human participants or human data. See also policy information about sex, gender (identity/presentation), and sexual orientation and race, ethnicity and racism.

Reporting on sex and gender	N/A
Reporting on race, ethnicity, or other socially relevant groupings	N/A
Population characteristics	N/A
Recruitment	N/A
Ethics oversight	N/A

Note that full information on the approval of the study protocol must also be provided in the manuscript.

Field-specific reporting

Please select the one below that is the best fit for your research. If you are not sure, read the appropriate sections before making your selection.

☒ Life sciences ☐ Behavioural & social sciences ☐ Ecological, evolutionary & environmental sciences

For a reference copy of the document with all sections, see nature.com/documents/nr-reporting-summary-flat.pdf

Life sciences study design

All studies must disclose on these points even when the disclosure is negative.

Sample size	No statistical methods were used to predetermine sample size for experiments, but sample sizes are in line with field standards. All experiments were performed at least twice.
Data exclusions	No data were excluded from this study.
Replication	All data are from a minimum of two independent experiments. All replication attempts were successful.
Randomization	Samples were not randomized in this study. Covariates were controlled by reproducing the experiments on separate days.
Blinding	We did not blind samples as, after each experimental setup, all measurements and analyses were performed identically across all conditions.

Reporting for specific materials, systems and methods

We require information from authors about some types of materials, experimental systems and methods used in many studies. Here, indicate whether each material, system or method listed is relevant to your study. If you are not sure if a list item applies to your research, read the appropriate section before selecting a response.

Materials & experimental systems

n/a	Involved in the study
☐	☒ Antibodies
☒	☐ Eukaryotic cell lines
☒	☐ Palaeontology and archaeology
☒	☐ Animals and other organisms
☒	☐ Clinical data
☒	☐ Dual use research of concern
☒	☐ Plants

Methods

n/a	Involved in the study
☒	☐ ChIP-seq
☒	☐ Flow cytometry
☒	☐ MRI-based neuroimaging

Antibodies

Antibodies used

Anti-VSVG (Sigma, V4888); anti-Rabbit IgG HRP (7074S, NEB); P. aeruginosa serotype O5 mouse monoclonal antibody (MediMabs, MM-76605-1; Clone ID MF15-4); anti-Mouse HRP (NEB, 7076S)

Validation

The Anti-VSVG antibody has been used numerous times to detect VSVG-tagged proteins by western blot and validation information can be found at the manufacturers' website (https://www.sigmaaldrich.com/US/en/product/sigma/v4888). In addition, we verified that the antibody does not react with Pseudomonas aeruginosa lysates derived from strains that do not encode a VSVG-tagged protein (see figure S2). P. aeruginosa serotype O5 mouse monoclonal antibody has been validated previously to detect O-antigen from P. aeruginosa serotype O5 (https://link.springer.com/protocol/10.1007/978-1-4939-0473-0_31).
