# Supplementary figures and images for: Synthesis of lipid-linked precursors of the bacterial cell wall is governed by a feedback control mechanism in Pseudomonas aeruginosa
Source: Nat Microbiol. 2024 Feb 9;9(3):763–75. doi: 10.1038/s41564-024-01603-2 (PMC10914600; doi:10.1038/s41564-024-01603-2)

Source Data: uncropped blot from Extended Data Figure 3B

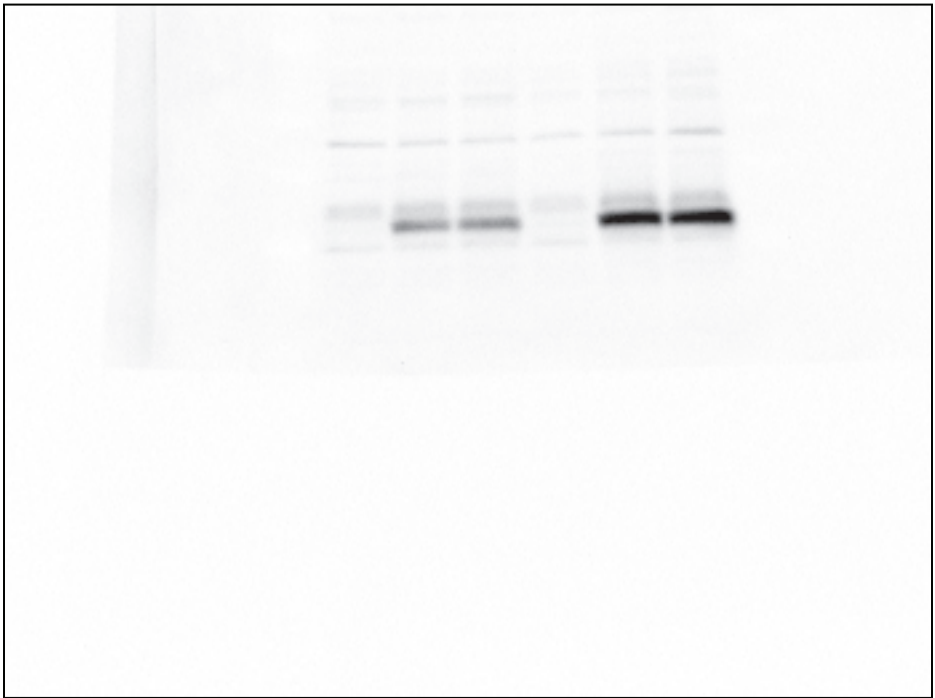

Supplement: Supplementary file 8 — Unprocessed western blots. [file 41564_2024_1603_MOESM8_ESM.pdf]

Source Data: uncropped blots from Extended Data Figure 5B

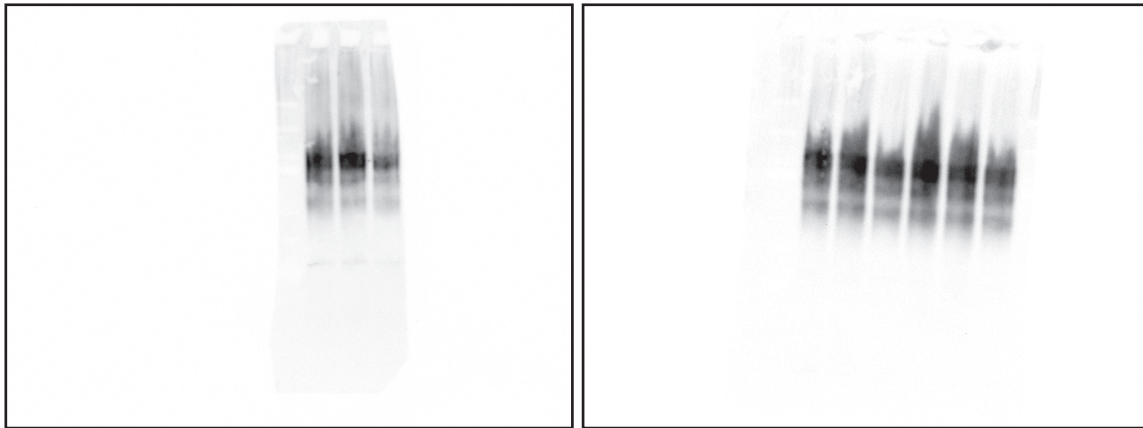

Supplement: Supplementary file 9 — Unprocessed western blots. [file 41564_2024_1603_MOESM9_ESM.pdf]
